# Clinical associates and access to healthcare in the Eastern Cape province of South Africa

**DOI:** 10.4102/phcfm.v14i1.3027

**Published:** 2022-03-28

**Authors:** Grace Isembatya, Aloysious Kakia, Jehu E. Iputo

**Affiliations:** 1 Department of Family Medicine and Rural Health, Faculty of Health Sciences, Walter Sisulu University, Mthatha, South Africa; 2 Department of Physiology, Faculty of Health Sciences, Busitema University, Tororo, Uganda; 3 Department of Medical Education, Walter Sisulu University, Mthatha, South Africa

**Keywords:** clinical associates, district hospitals, healthcare access, supervision, Eastern Cape, task shifting

## Abstract

**Background:**

Clinical associates (ClinAs) were introduced into South Africa as part of the remedy for the severe shortage of healthcare workers in rural areas. Walter Sisulu University (WSU) graduated 100 ClinAs between 2011 and 2014. These ClinAs were expected to be based at district hospitals where they would work under the supervision of doctors, reduce the workload of doctors and increase access to healthcare in the Eastern Cape.

**Aim:**

This study aimed to examine the role played by ClinAs in healthcare delivery in Eastern Cape district hospitals, and to determine whether the training of ClinAs adequately prepared them for this role.

**Setting:**

The study was conducted in the Eastern Cape province of South Africa amongst ClinAs who graduated from WSU between 2011 and 2014, and healthcare workers from Madzikane KaZulu Memorial Hospital.

**Methods:**

This was an exploratory cross-sectional mixed methods study with a convergent design. Surveys and in-depth interviews were conducted amongst ClinAs, nurses, doctors and one pharmacist. Both qualitative and quantitative data were analysed and reported.

**Results:**

Clinical associates are seen to improve the workload of doctors, and to increase access to healthcare. Thirty-four percent of ClinAs were no longer contributing to healthcare in the Eastern Cape.

**Conclusion:**

Clinical associates are making a significant contribution to access to healthcare in the Eastern Cape. Their supervision regimen needs to be revisited and factors that contribute to the attrition of ClinAs in the Eastern Cape need to be addressed.

## Introduction

Clinical associates (ClinAs) are mid-level healthcare workers (MLHWs) in South Africa who were introduced into the workforce as part of a strategy to remedy the shortage of health manpower at district hospitals.^1^ Training of this cadre began in 2008 and is conducted at Walter Sisulu University (WSU), University of the Witwatersrand and University of Pretoria, as a 3-year Bachelor of Clinical Medical practice (BCMP) course. Clinical associates in South Africa are regulated by the Health Professions Council of South Africa (HPCSA), and their role is defined in the *South Africa Health Professions Act* 1974 under the regulations defining the scope of practice of ClinAs.^2^ At the district hospital, ClinAs are expected to take on some of the tasks typically performed by doctors, thus reducing doctors’ workload and increasing access to healthcare.^1^ This is in line with World Health Organization (WHO) guidelines on task shifting. The WHO describes task shifting *as*

[…] The rational redistribution of tasks among health workforce teams, [*whereby*] specific tasks are moved, where appropriate, from highly qualified health workers to health workers with shorter training and fewer qualifications in order to make more efficient use of the available human resources for health. (p. 7)^3^

According to the ClinA scope of practice, a ClinA who has practised for a continuous period of less than two years must be under continuous and hands on supervision, and work in the same clinical setting as the supervising doctor. The same Act states that ClinAs who have worked for 2–4 years must report to the supervising doctor after every task and have their management plans and decisions countersigned by the supervisor. After practising for five continuous years, a ClinA may practice independently on a day-to-day basis and does not have to report to the supervising doctor.^2^ The supervision of ClinAs is aimed at maintaining a high quality of healthcare while increasing access through the process of task shifting.

The use of MLHWs that are equivalent to ClinAs has boosted the efficiency of healthcare service delivery on the African continent.^4,5,6^ Several benefits of having ClinAs in the healthcare system in South Africa have been reported too. To mention a few, Bac et al.^7^ found that using ClinAs in a rural district hospital led to improvement in quality of patient care by reducing waiting times in casualty and outpatient departments (OPD) because the ClinAs took on much of the workload of the medical team. Hamm et al.^8^ looked at the cost of training and employing ClinAs in South Africa and found that the ClinAs were two and a half times less costly to train than doctors, and about three times less costly to employ. In the same study, ClinAs were found to free up the time of doctors by 50% – 70% while providing the same quality of care.^8^ Clinical associates are, therefore, a means of meeting the ever-increasing demand for rural healthcare workers.

A total of 100 ClinAs graduated from WSU between 2011 and 2014 who were deployed at district hospitals in the Eastern Cape province. It was expected that the ClinAs would relieve doctors of the heavy workload they were experiencing and expand access to primary health care services.^9^ They were also expected to work under the supervision of doctors for the mandatory 5-year period. Information regarding whether the ClinAs were living up to these expectations has been in short supply. This study was, therefore, conducted to examine the role played by ClinAs in increasing access to healthcare in Eastern Cape district hospitals. Specifically, the study sought to determine the perceptions regarding the adequacy of ClinA training at WSU, to find out whether ClinAs are perceived to have led to an increase in access to care at district hospitals in the Eastern Cape and to find out about the extent of supervision of ClinAs.

## Research methods and design

### Study design

This was an exploratory cross-sectional mixed methods study with a convergent design. The convergent design was used because it involves collecting and analysing both qualitative and quantitative data and then merging them for the purpose of comparing or combining the results, thus gaining a better understanding of the topic under study.^9^ It also has the advantage of bringing together the strengths of both the qualitative and quantitative methods.^9^ This study was conducted in the Eastern Cape province of South Africa in mainly rural districts.

### Study population and sampling strategy

Participants were drawn from two sources: ClinAs WHO graduated from WSU between 2011 and 2014 and were working at district hospitals in the Eastern Cape, and healthcare workers from Madzikane KaZulu Memorial (MKM) Hospital that regularly interacted with ClinAs in the workplace. The MKM hospital was selected for the study because it is a typical rural district hospital that has had ClinAs since they were first deployed in 2011. It is situated in Alfred Nzo district municipality of the Eastern Cape province.

The staff who participated in the study at MKM Hospital included doctors, the nurses who worked in OPD and casualty departments and a pharmacist. The number of doctors at MKM Hospital at the time of data collection was 11, the nurses in OPD and casualty departments were 30 and there was one pharmacist, making a total of 42 staff. Five of these staff participated in key informant interviews. The remaining 37 were invited to participate by completing a survey questionnaire.

The in-depth interviews at MKM Hospital were conducted with staff who had worked closely with ClinAs. To this end, a total of five interviews were conducted. These included three medical officers (doctors), the nurse in charge of OPD and the Clinical manager (a doctor). The three medical officers were chosen because they mainly worked in OPD where ClinAs in MKM Hospital are based, and are expected to have supervisory oversight of the ClinAs. The nurse in-charge of OPD was interviewed based on the fact that she worked daily with the ClinAs as she oversaw OPD activities. The clinical manager was interviewed because he was responsible for overseeing the clinical function of the hospital including the ClinAs in OPD.

The ClinAs who graduated from WSU between 2011 and 2014 were traced through snowballing. Those whose whereabouts were known provided information and telephone contacts of others, who in turn provided contact information of those who had not yet been contacted. Ninety-five out of the 100 graduates were successfully traced and their distribution is shown in Table 1.

**TABLE 1:** Distribution of 2011–2014 Walter Sisulu University clinical associate graduates.

Status	Frequency	Percentage
Working in district hospitals in Eastern cape	55	55
Enrolled in MBChB programmes	34	34
Working for non-governmental organisations in Eastern Cape	5	5
Deceased	1	1
Could not be traced	5	5

**Total**	**100**	**100**

MBChB, Bachelor of Medicine and Bachelor of Surgery.

### Data collection

Data were collected using three instruments: a semi-structured self-administered paper-based survey questionnaire for MKM hospital staff; a semi-structured self-administered paper-based survey questionnaire for the ClinAs at district hospitals and a semi-structured interview guide for key informant interviews. The survey questionnaires and the key informant interview guide were developed based on the literature and the objectives of the study. They inquired about the competence of the ClinAs, strengths and weaknesses of ClinA training, units the ClinAs work in, how ready they felt ClinAs were when they first started working, supervision of ClinAs by doctors, procedures performed by ClinAs, involvement in patient care and contribution to human immunodeficiency virus (HIV) and tuberculoses (TB) care amongst other issues.

Survey questionnaires for the MKM Hospital staff were delivered by the first author and followed up after some days. Seven out of the 11 doctors were given questionnaires. The other four doctors participated in the in-depth interviews as key informants. Twenty-nine nurses received questionnaires and one nurse participated in the in-depth interviews. Appointments were made for key informant interviews which were then conducted in offices at the hospital administration building, ensuring that confidentiality was maintained. The interviews were audio-recorded after permission was sought from the participants. Survey questionnaires were given to the 55 ClinAs who were working at district hospitals through hand delivery, where possible, or by e-mail for those who were far and had provided their email addresses. Data were collected in November 2015 and December 2015.

### Data analysis

Data from the survey questionnaires were coded and then entered into an Excel spreadsheet. The quantitative data were mainly analysed using descriptive statistics. The comparison between the responses of ClinAs and those of the MKM hospital staff was done using student’s *t*-test. The responses to open-ended questions in the survey questionnaires regarding the strengths and weaknesses of the WSU training programme were coded and then put into categories. The frequency of responses in each category was then determined.

The key informant interviews were transcribed verbatim by a third party. The transcripts were then reviewed by the first author and compared with the audio-recordings, and then confirmed by the second author. Corrections made were necessary so as to ensure accuracy of the transcription. Data were analysed using the six steps of thematic analysis as described by Braun and Clarke.^10^ These were: familiarisation with the data, generating initial codes, searching for themes, reviewing the themes, defining and naming the themes and producing the report. To this end, familiarisation was achieved by listening to the audio-recordings and checking that the transcription was accurate, and reading through the transcribed scrips several times before coding. The data were then assigned initial codes while keeping an open stance to allow for modification of codes as the analysis proceeded. Categories were developed from the codes that had a relationship with each other and these were then built into themes. All three authors were involved in the process of qualitative data analysis so as to enhance validity. The four criteria for trustworthiness of qualitative data were adhered to. Credibility and dependability have been assured through triangulation; transferability has been enhanced by the thick description in the key informant narratives and confirmability was enhanced through supervisory oversight by the third author.

### Ethical considerations

Ethical approval was provided by Walter Sisulu University Research Ethics Committee (protocol number 017/15). Permission was also sought from the Eastern Cape province Department of Health (Protocol number EC−2015RP34−458) and Madzikane KaZulu Memorial hospital management. Informed consent was obtained from each of the participants.

## Results

The results of this study are presented under two main sections. The quantitative results of the survey of MKM Hospital staff and the survey of ClinAs working in Eastern Cape district hospitals are presented first, followed by the qualitative results of the in-depth key informant interviews conducted at MKM Hospital.

### Survey respondent characteristics

Table 2 shows the questionnaires distributed and the number returned. The overall response rate was 76%.

**TABLE 2:** Madzikane KaZulu Memorial hospital staff survey respondents by cadre.

Cadre	Number distributed	Number returned	Response rate (%)
Medical doctor	7	7	100
Professional nurse	15	12	80
Pharmacist	1	1	100
Other nurses (enrolled nurse and nursing assistant)	14	8	57

**Total**	**37**	**28**	**76**

Fifty percent of the MKM hospital staff who responded had worked in the hospital for at least three years, and 79% had worked for at least one year. These respondents had, therefore, worked with ClinAs for a period sufficient enough to make judgement. Of the 55 questionnaires that were sent to ClinAs working at district hospitals, 40 were returned, giving a response rate of 73%.

### Survey results

The surveys addressed the areas of perceived adequacy of ClinA training, ClinAs’ role in increasing access to healthcare and the supervision of ClinAs. The results are reported under these headings.

#### Perceived adequacy of clinical associates training

The adequacy of ClinA training was looked at from the point of view of participants’ perceptions of ClinA competency and their perceptions of the strengths and weaknesses of the WSU ClinA training programme. Survey respondents were asked to rate the ClinAs’ competence on a scale of one to six, one being very poor and six being excellent. The rating was based on clinical work readiness at the time of employment, communication skills, ability to function as a team member, motor skills, professionalism and preparedness to work in underserved rural communities. The results are shown in Table 3.

**TABLE 3:** Rating of clinical associate competencies by clinical associates in Eastern Cape and Madzikane KaZulu Memorial Hospital staff.

Area of competency	Eastern Cape ClinA responses				MKM Hospital staff responses				*p*
	Average	s.d.	Lowest	Highest	Average	s.d.	Lowest score	Highest score	
Clinical work readiness	4.12	0.94	1.00	6.00	5.00	0.76	4.00	6.00	0.1434
Communication skills	4.92	0.97	2.00	6.00	5.52	0.58	4.00	6.00	0.0053
Function as a team member	5.40	0.81	3.00	6.00	5.47	0.79	3.00	6.00	0.4620
Overall motor skills	4.77	0.86	1.00	6.00	4.89	0.73	4.00	6.00	0.2415
Professionalism	5.69	0.57	1.00	6.00	5.52	0.58	4.00	6.00	0.4570
Preparedness to work in rural communities	4.62	1.10	4.00	6.00	5.45	0.72	4.00	6.00	0.0168

ClinA, clinical associate; s.d., standard deviations.

The MKM hospital staff rated the ClinAs highest for their communications skills and professionalism, and lowest for motor skills. The ClinAs rated themselves highest for professionalism, and lowest for clinical work readiness at the time of employment. The difference in the means was significant for preparedness to work in rural communities and communication skills. For both criteria, the hospital staff rated the ClinAs higher.

Participants were asked to state what they thought were the major strengths and weaknesses of the WSU ClinA training programme. Multiple answers were allowed for these open-ended questions. In order of frequency, the MKM staff reported three major strengths: Training ClinAs in rural district hospitals gives them opportunity to get used to the environment where they will work after graduating; the graduates have a positive attitude towards work; the graduates are well-prepared to work with patients. The ClinA respondents reported the following strengths in order of descending frequency: They were exposed to many clinical procedures and patients during training; the curriculum is comprehensive and covers many aspects of learning required for clinical work; early exposure to clinical environment gave them experience and confidence in clinical work; there are committed WSU tutors assigned to each training hospital site.

With regard to the weaknesses of the programme, the MKM hospital respondents reported three weaknesses with equal frequency: the basic science knowledge is taught superficially at the University; some ClinAs lack the knowledge or self-confidence to perform emergency procedures; the prescriptions of the graduates need to be countersigned by doctors. On the other hand, the major weaknesses reported by ClinAs were: the period for basic science lectures was too short for students to grasp all that was being taught, therefore, the knowledge of basic sciences is insufficient; there is a lack of uniformity in the way teaching is done at the different district hospital training sites; there was a lack of learning resources such as good textbooks and internet connection at district hospital training sites; some district hospital training sites suffered shortage of WSU tutors making it difficult for the one available tutor to cope.

#### Clinical associates’ role in increasing access to healthcare

In order to assess the perceptions as to whether ClinAs increase access to healthcare, survey respondents were asked about their views on whether ClinAs relieve doctors of some of their clinical responsibilities, ClinAs involvement in TB and HIV care and ClinAs performance of therapeutic and investigative procedures typically done by doctors.

The survey respondents at the MKM hospital were asked to indicate whether they strongly agreed, agreed, disagreed, strongly disagreed or were not sure that the ClinAs have relieved doctors of some clinical responsibilities. Fifty percent strongly agreed and 47% agreed, whilst 3% were unsure. None of the participants disagreed.

The ClinAs were asked to list the procedures that they perform which are typically done by doctors. These are shown in Table 4. Multiple responses were accepted for this question.

**TABLE 4:** Clinical procedures performed by clinical associates that are typically done by doctors.

Procedure	Percentage
Lumbar puncture	72
Pleural tap	54
Inserting intercostal chest drainage	44
Ordering and interpreting of findings of investigative procedures such as X-ray, basic blood investigations, urinalysis	28
Perform incision and drainage	23
Perform ascites taping	23
Inserting intravenous line	15
Perform circumcision	15
Administering spinal anaesthesia	13
Perform suturing of cut wounds	10
Drawing of blood samples	10

Lumbar puncture, pleural tap and inserting intercostal chest drains came at the top with over 40% of ClinAs reporting that they do these procedures. Procedures that were mentioned by less than 10% of ClinAs and are not shown in Table 6 included performing ultrasound, taking pus swab, reduction of dislocation and fractures and applying plaster of paris, performing fine needle aspiration, ear syringing, suprapubic catheterisation, joint aspiration, blood transfusion, skin biopsy, hydrocele taping, intraosseous infusion, assessing and referring complicated cases.

Clinical associate respondents were asked which units they work in at the Eastern Cape district hospitals where they are based. They reported that they mostly work in the outpatients’ department followed by casualty/trauma department as shown in Table 5.

**TABLE 5:** Units at Eastern Cape District Hospitals where clinical associates work.

Hospital unit	Percentage
Outpatient	98
Trauma/casualty	55
Medicine	40
Paediatrics	30
Maternity/gynaecology	25
Theatre	18
Surgical	18
HIV/AIDS clinic	18
TB clinic/ward	10

TB, tuberculosis; HIV, human immunodeficiency virus; AIDS, acquired immunodeficiency syndrome.

#### Supervision of clinical associates

Clinical associates were asked to indicate the extent to which they are supervised by a doctor as they do clinical work. Fifty-five percent of the ClinAs indicated that they work autonomously with no direct supervision from a doctor, whilst 32.5% of them indicated that they are supervised sometimes. Five percent of them are supervised most of the time and 7.5% of them are always supervised. Participants who did not indicate that they are supervised ‘always’ were asked to indicate what the doctors do at the time when they are not being supervised. Ninety-five percent of them indicated that the doctors are busy with other activities and therefore could not supervise them fully, whilst 5% of them indicated that they are not always supervised because there were no doctors at their hospitals.

### Key informant interviews

Analysis of the key informant interviews resulted in three themes: Adequacy of ClinAs’ training, role of ClinAs in increasing access to care and supervision of ClinAs. The results are presented here under each of the themes.

#### Adequacy of clinical associates’ training

Clinical associates were seen as good team players who depict a high level of professionalism:

‘What is important in the district hospital more than anything is the unity that people should have to work together … the clinical associates are actually more united with us than some of us doctors here.’ (Doctor 1, Nov 2015)

‘I have only worked with one clinical associate but she is very professional, comes to work on time, sees the patients, leaves work when she is supposed to leave work, very efficient, works very hard with the patients and also deals well with the colleagues. So she is, well, she has a good conduct.’ (Doctor 1, Nov 2015)

The skills of the ClinAs are said to improve with time, particularly the prescribing skills:

‘I think when they start it’s a matter of doses and all those things, although the drug names they know it but doses, the duration, they are a challenge. As soon as they get clinical exposure they start to acquire that.’ (Doctor 3, Nov 2015)

Clinical associates’ clinical reasoning and decision-making skills were commended:

‘For clinical associates I would commend the program when it comes to clinical reasoning. In fact, for the clinical associates that I have been able to associate with are very sound at reasoning and they are able to diagnose properly more particularly chronic and acute conditions.’ (Doctor, clinical manager, Nov 2015)

#### Clinical associates’ role in increasing access to healthcare

Key informants were asked whether ClinAs increase access to healthcare. Unanimously, having ClinAs at this district hospital is seen to increase the number of patients who access healthcare services at the OPD:

‘With the addition of a clinical associate, let’s say there are three doctors that are posted in OPD and then each doctor will see let’s say 25 patients a day. If there is no clinical associate there will just be 75 patients seen a day, but with a clinical associate there, 100 patients will be seen a day because she will also handle 25 patients on her own. So in that way access to health care is more and the patients have access now, much better than before.’ (Doctor 1, Nov 2015)

‘At least we don’t see a number of patients sleeping over. Yah the turnover is good.’ (Doctor, 2 Nov 2015)

‘Yes, you know we have shortage of the doctors in the hospital, so the presence of the clinical associates was of good.’ (Nurse in charge OPD, Nov 2015)

At MKM hospital, ClinAs’ contribution to increasing access to care is confined to the outpatients’ department:

‘Clinical associates are mostly confined to the OPD, we had not been able to utilise them for ward and casualty assignments because we have been experiencing shortage of doctors for quite some good time.’ (Doctor, clinical manager, Nov 2015)

Clinical associates were reported to increase access to TB and HIV care. This is done at the OPD as part of integrated services and not in specialised clinics:

‘Yes. By the mere presence of them being in OPD and attending to such patients, yeah. Like all of us, we’ll initiate HAART [*highly active antiretroviral therapy*], we will request patients to do HCT (HIV counselling and testing), so in that way they do play a role, a huge role. in fact, clinical associates here are like doctors, nothing like you are a clinical associate, you can’t do this, you can’t do that, she just sits in that room and sees patients like all of us.’ (Doctor, 3 Nov 2015)

‘Yes, what they do is that they send the clients for screening and then the patients do come back to collect their results … and then they initiate them on ARVs [*antiretroviral therapy*] and they do take some bloods.’ (Nurse in charge OPD, Nov 2015)

‘Yes within their scope of training, clinical associates with whom we have worked with are able to perform well with identification of TB … which is diagnosis and its management. Where they have challenges in that effect they would consult senior doctors and they would be in that position to assist clinical associates.’ (Doctor, clinical manager, Nov 2015)

‘they do the same [*for TB*] as in HIV/AIDS: counselling, diagnosis, treatment.’ (Doctor 2, Nov 2015)

Key informants reported that ClinAs relieve doctors of their heavy workload:

‘They have been able not only to relieve the duty of doctors but a relief to shortage of medical personnel in this hospital.’ (Doctor, clinical manager, Nov 2015)

‘They have performed a lot of procedures such as administering spinal anaesthesia, assisting in theatre and we haven’t heard any negative comment about those procedures because they do admit patients, they do Lumbar puncture, do help as they discuss patients with Nelson Mandela tertiary hospital specialists.’ (Doctor, clinical manager, Nov 2015)

Relieving the doctors of some tasks allows them (doctors) to attend to clinical duties within other hospital departments:

‘Sometimes even the doctors that are placed in OPD have to go to theatre or attend casualty so you don’t have to be really worried because there is a clinical associate that is there who is attending to [*the patients*].’ (Doctor 1, Nov 2015)

#### Supervision of clinical associates

Key informants were asked about the extent to which ClinAs are supervised. The responses indicate that there is minimal supervision of ClinAs:

‘… truly speaking, sometimes they [*ClinAs*] do come alone and do all of these responsibilities alone. May be the doctors are busy in the other wards.’ (Nurse in charge OPD, Nov 2015)

‘With our arrangement here, the doctors also have different assignment when the clinical associates have to take responsibilities …’ (Doctor, clinical manager, Nov 2015)

‘… the question of supervision, again, is a big one. Like I said we are short staffed ourselves.’ (Doctor 2, Nov 2015)

‘I said you hardly go to that space of direct supervision, to actually see how a certain examination is performed. Because of shortage of staff we hardly, like, be in the same consulting room to witness those examinations.’ (Doctor 3, Nov 2015)

The ClinAs, however, make an effort to consult the doctors whenever they are faced with challenges in their clinical work:

‘Like what I have done here in my capacity as a clinical manager was to place the doctors to the OPD, sometimes its three doctors then clinical associates make 4 and she will be able to see patients. Where she has challenges she will be able to consult with the more senior doctors available at that time, and sometimes when she is available any time in the vicinity, she comes around so as to discuss patient matters with me.’ (Doctor, clinical manager, Nov 2015)

Furthermore, the doctors said that the frequency of consulting doctors is higher when the ClinAs have just started clinical practice, but with time they grow in competence and the frequency decreases.

## Discussion

### Adequacy of clinical associates’ training

The training of ClinAs at WSU is perceived to be adequate as evidenced by the high rating given for the ClinA competencies by both ClinAs and MKM hospital staff. This view is backed by the key informants who saw ClinAs to be highly professional, having sound clinical reasoning and being good team players. The training of ClinAs in rural district hospitals is seen as a great strength of the programme because it gives them the opportunity to learn from the environment where they will work after graduating and gives them an early and wide experience with patients, which builds their confidence. The presence of a dedicated tutor at each hospital training site is considered a plus for the programme in addition to having a comprehensive curriculum. The strengths mentioned by the ClinA respondents all centre around the fact that the ClinA training is based at district hospitals in a distributed model of health professionals’ education. De Villiers et al.^11^ state that distributed health professional education has the potential benefits of providing opportunities for learner exposure to various facets, including the local health context, the continuum of comprehensive care, the role of context in health and illness and having the potential to address the problem of mal-distribution of health manpower. These benefits are being realised in the WSU ClinA training. Literature on the competence of ClinAs is also in consonance with the current findings. In a sample of 4850 patient files analysed retrospectively, Ngcobo et al.,^12^ found that ClinAs in Tshwane district in South Africa performed voluntary male medical circumcisions at a clinical standard that is comparable to circumcisions performed by doctors. Parle, Ross and Doe^13^ in England also report a standard of care by physician assistants (the equivalent of ClinAs) that is similar to that of doctors working in primary care. Furthermore, these results agree with the findings of the systematic review by Wilson et al.^4^ that showed that clinical officers (ClinA equivalents) were as competent as doctors in performance of caesarean sections.

Teaching of the basic sciences was seen to be a major weakness of the programme by both the ClinAs and MKM hospital staff. The depth and the time allocated to basic sciences were perceived to be insufficient. The fact that this was noticed by the hospital staff surveyed indicates that it is an evident gap that needs to be addressed. Cox and Simpson^14^ state that nursing students’ self-efficacy is affected by the extent to which they believe that they understand the basic sciences. The perceived insufficient coverage of the basic sciences in this programme could potentially lead to self-efficacy issues amongst ClinAs. The issue of self-efficacy is further highlighted in this study by the observation by MKM hospital staff that some ClinAs lack the confidence to perform emergency procedures. Self-efficacy has been shown to be positively related to the performance of clinical procedures by healthcare workers^11^ such as the emergency procedures mentioned by the staff. The fact that the ClinAs generally rated their competence lower than the hospital staff rated them may also be an indicator of low self-efficacy. The literature states that self-efficacy improves with the duration of experience in the clinical setting.^15,16^ In this study, this is reflected in the finding that there is a reduced frequency of ClinAs consulting doctors with time.

### Increasing access to healthcare

In line with the purpose of the ClinA programme in South Africa, the ClinAs are seen to increase access to healthcare. They are an extra hand in the OPDs, where patient lines and waiting times tend to be long. Both the quantitative and qualitative analyses agree that taking on tasks that are typically done by the doctors allows the doctors to pay attention to the more seriously ill patients and also increases the number of patients seen at the hospitals. Taking on the doctors’ tasks is enhanced by ClinAs’ ability to perform a wide array of clinical procedures as shown in Table 4 and also reported by the ClinAs as a major strength of the training programme. The ClinAs increase access to HIV and TB care due to their role in diagnosis, treatment and counselling at the OPDs. Their role in follow-up of TB and HIV patients is minimal as this is done at the lower-level health units mainly by community healthcare workers. These study results are comparable to the findings of Dambisya and Matinhure^6^ regarding clinical officers in Uganda, and Ngcobo et al.^11^ who showed that ClinAs in Tshwane district helped to meet the high demand for medical male circumcisions. This is the evidence that task shifting from doctors to ClinAs in the Eastern Cape province has been realised in the hospitals that have ClinAs. Clinical associates’ role in inpatient management is limited. The essence of this is that ClinAs have found a niche in the OPD and has implications for training because they need to be well-prepared for this niche.

As an incidental finding of this study, 34% of the ClinAs in the 2011–2014 cohort from WSU had enrolled for Bachelor of Medicine and surgery programmes at various institutions at the time of data collection. This represents a loss of 34% of manpower that the ClinA profession would have offered the rural population of the Eastern Cape. At the advent of deployment of ClinAs in South African hospitals, Doherty, Couper and Fonn^17^ identified the possibility of brain drain resulting from the absence of a clear career path for ClinAs as one of the key challenges that need to be addressed in order for the health system to realise the full potential of ClinAs. Ngcobo^18^ asserts that ClinAs are very likely to leave the profession because of the lack of support from the National Department of Health, lack of a clear career path, lack of a scope of practice earlier in the profession and being overworked and underpaid. The attrition seen in this study confirms the fears raised by these authors and is a dent in the contribution of ClinAs to access to healthcare in the Eastern Cape.

### Supervision of clinical associates

At the time of data collection, the first cohort of ClinAs had been working for just four years. None of the participants had reached the ‘more than five years’ prescribed for working independently. The lack of supervision of ClinAs in the Eastern Cape, therefore, represents a breach of the act governing their practice. As the purpose of supervision is to maintain a high quality of healthcare provided, this raises a concern about the possible compromise of quality of healthcare. The main driver of poor supervision is the workload on the doctors which makes direct supervision not practical. Another possible explanation is that after a short period of observation, the district hospital staff develop a confidence in the ClinAs and allow them to work independently. This situation begs for a review of the supervisory regimen for ClinAs more so because they are seen to be competent in spite of the lack of supervision, yet, theoretically at least, the quality of healthcare is at stake. On a positive note, the ClinAs are reported to consult whenever they feel the need to do so, and their skills are said to improve over time.

## Study limitations

The major limitation of this study is that the non-ClinA participants were from only one hospital. Involving non-ClinA healthcare workers from other district hospitals in the Eastern Cape could have potentially provided a more comprehensive picture. In this study however, we made an effort to involve all the ClinAs who we could get in contact with from the various hospitals in the Eastern Cape so as to have the broad picture from the province. The key informant interviews and survey at MKM hospital then provided in-depth information and also confirm the data gathered from ClinAs in the province because they were conducted with participants who work closely with ClinAs.

## Conclusion

This study has demonstrated that the ClinA training programme is perceived to have adequately prepared the ClinA graduates for the role they play in healthcare delivery in the Eastern Cape province. The ClinAs have increased access to care in the Eastern Cape by taking on tasks typically performed by doctors and by increasing the number of clinicians available at district hospitals, thus making the ClinAs an important player for task shifting in the Eastern Cape. There is a glaring gap in the supervision of ClinAs that needs to be addressed by the Eastern Cape Department of Health and the HPCSA. A 1-year internship should be considered so as to ensure at least a year of adequate supervision of ClinAs. The contribution of ClinAs to increasing access to healthcare is dented by the high rate of attrition from the profession. This attrition needs to be investigated and remedied for the Eastern Cape to realise the full benefit of having ClinAs at district hospitals.
